# Transcriptome profiling of ontogeny in the acridid grasshopper *Chorthippus biguttulus*

**DOI:** 10.1371/journal.pone.0177367

**Published:** 2017-05-17

**Authors:** Emma L. Berdan, Jonas Finck, Paul R. Johnston, Isabelle Waurick, Camila J. Mazzoni, Frieder Mayer

**Affiliations:** 1 Museum für Naturkunde Berlin, Leibniz Institute for Evolution and Biodiversity Science, Berlin, Germany; 2 Behavioural Physiology, Department of Biology, Humboldt-Universität zu Berlin, Berlin, Germany; 3 Freie Universität Berlin, Berlin, Germany; 4 Berlin Center for Genomics in Biodiversity Research, Berlin, Germany; 5 Leibniz-Institut für Zoo- und Wildtierforschung (IZW), Berlin, Germany; 6 Berlin-Brandenburg Institute of Advanced Biodiversity Research (BBIB), Berlin, Germany; USDA Agricultural Research Service, UNITED STATES

## Abstract

Acridid grasshoppers (Orthoptera:Acrididae) are widely used model organisms for developmental, evolutionary, and neurobiological research. Although there has been recent influx of orthopteran transcriptomic resources, many use pooled ontogenetic stages obscuring information about changes in gene expression during development. Here we developed a *de novo* transcriptome spanning 7 stages in the life cycle of the acridid grasshopper *Chorthippus biguttulus*. Samples from different stages encompassing embryonic development through adults were used for transcriptomic profiling, revealing patterns of differential gene expression that highlight processes in the different life stages. These patterns were validated with semi-quantitative RT-PCR. Embryonic development showed a strongly differentiated expression pattern compared to all of the other stages and genes upregulated in this stage were involved in signaling, cellular differentiation, and organ development. Our study is one of the first to examine gene expression during post-embryonic development in a hemimetabolous insect and we found that only the fourth and fifth instars had clusters of genes upregulated during these stages. These genes are involved in various processes ranging from synthesis of biogenic amines to chitin binding. These observations indicate that post-embryonic ontogeny is not a continuous process and that some instars are differentiated. Finally, genes upregulated in the imago were generally involved in aging and immunity. Our study highlights the importance of looking at ontogeny as a whole and indicates promising directions for future research in orthopteran development.

## Introduction

Acridid grasshoppers (Orthoptera: acrididae) are a model system for acoustic communication research [1, 2] and are widely used for studies of speciation, sexual selection, neurobiology (particularly acoustic neurobiology), and development [3–9]. One of the most well-studied Acrididae is *Chorthippus biguttulus* which has been used in studies of acoustic communication [6, 10–12], development [13–15], and sexual selection [16, 17].

The emergence of next generation sequencing (NGS) techniques has drastically altered the face of biology. Platforms such as Illumina HiSeq, Roche 454, ABI SOLiD, and others can generate genomic or transcriptomic data rapidly and without any prior genomic resources [18, 19]. These techniques have been used to establish genomic resources for a subset of orthopteran species (including a single Acridid species) such as *Locusta migratoria* [20, 21], *Gryllus bimaculatus* [22], *Laupala kohalensis* [23], *Teleogryllus oceanicus* [24], *Chorthippus biguttulus* [25], *Gryllus rubens* [26], and *Schistocerca gregaria* [27]. These are useful genetic resources for searching for candidate genes but the ubiquitous pooling of tissues and developmental stages does not allow for information about timing and location of gene expression [done in all studies except 24, 26]. Additionally, several of these transcriptomes, such as the one currently available for *C*. *biguttulus*, only contain a single life stage. Separation of developmental stages is particularly important for determining the genetic basis of adaptive or divergent traits, as traits develop at distinct times during ontogeny.

**Fig 1:**
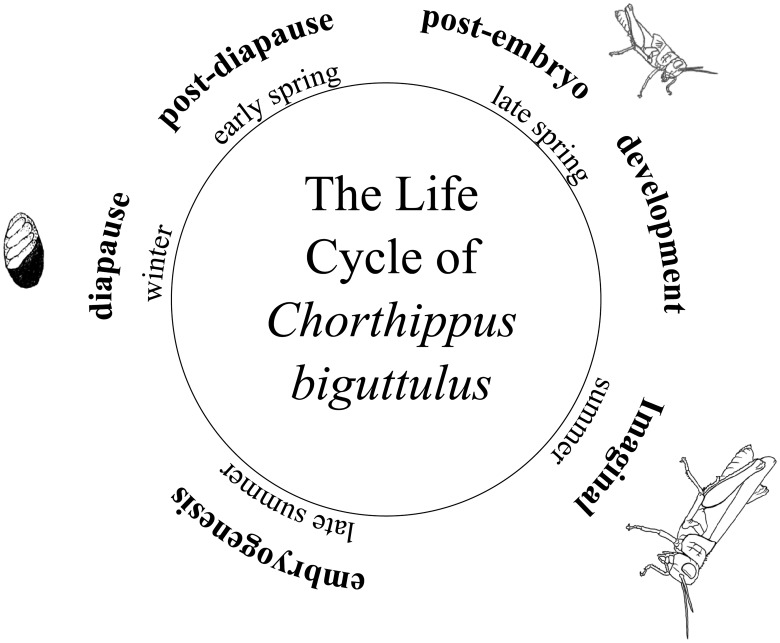
Life cycle of *Chorthippus biguttulus* indicating developmental stages and seasonal timing.

*Chorthippus biguttulus* are hemimetabolous meaning that ontogeny consists of three stages: the egg, the larval, and the imago (or adult). Unlike holometabolous insects, there is no pupal stage and nymphal stages resemble the imago [28]. Female *C*. *biguttulus* lay their ootheca, which consist of 7–10 eggs and a sticky secreted coating, in sandy and loose soil [29, 30]. Embryogenesis in Acrididae proceeds until the end of the mesentrepses or early blastokinesis when embryogenesis stops and diapause occurs [31]. Physiological activity in the embryo is diminished during diapause, which lasts from late summer to the early spring and protects the embryogenic process from unfavorable climate conditions. Hatching occurs in a brief temporal window in summer to coincide with optimal postembryonic development conditions and to synchronize ontogeny to maximize the number of fertile adults present at one time [Fig 1, 32, 33, 34]. In the lab hatching occurs after a 14 day post-diapause at 24°C [13]. Postembryonic development time in *C*. *biguttulus* is highly temperature dependent and is segmented in 4–5 instar stages [29, 35]. At 28 (±2)°C there are 4–7 days between the moltings that define the different instar stages (J. Finck, unpublished data). Throughout these instar stages morphological differentiation of the external reproductive organs and the wing shape occurs [36]. After the imaginal molt, the wings are fully formed and the gonads mature within 4–8 days [29].

Here we assemble and annotate a *de novo* transcriptome for *C*. *biguttulus* containing 7 different ontogenetic stages including embryonic and post-embryonic development. Most developmental research on arthropods has traditionally concentrated on embryonic development leaving the post-embryonic neglected [37]. We map individual stages back to our transcriptome to determine stage specific expression patterns. We identify 15 different gene expression clusters that reflect the unique processes that occur during ontogeny. In addition, we identify candidate genes that may play important roles at each ontogenetic stage.

## Materials and methods

### Animals

Late instar nymphs of *Chorthippus biguttulus* were collected at Wendebachstausee near Göttingen, Germany (N51°28’10.41, E9°56’24.98) in July and August of 2012. *Chorthippus biguttulus* is not protected in Germany. All animals were caught on public land which was free to enter. Therefore, neither access nor collection permissions were required. Grasshoppers were transferred to the lab and kept in mesh polyester cages (47.5 x47.5x93 cm, bugdorm Taichung, Taiwan). Animals were reared on a 16:8 h light:dark cycle and maintained at a temperature of 25–30°C with a humidity of 25–30%. They were fed ad libitum with a variety of grasses (*Festuca rubra rubra*, *Dactylis glomerata*, and *Poa pratensis*). All cages contained a plastic cup filled with moist granulate (Vermiculite Dämmstoffe, Germany) in which females could oviposit. Eggpods were transferred to petri dishes filled with moist granulate and incubated at room temperature for 4 weeks before diapause was simulated for 3–4 months at 4°C. During post-diapause the eggpods were kept at a temperature of 26 (±3)°C until hatching. We collected 5–9 individuals from 6 different developmental stages. Nine eggs from 2 eggpods were frozen in liquid nitrogen 7 days post-diapause. Eight individuals from the first instar stage were taken 2 days after hatching and an additional 8 were sampled two days after the first molt. An additional 5 individuals were frozen at 1–5 days post-molt for the 3^rd^, 4^th^, and 5^th^ instars. All individuals were pooled by stage (resulting in 6 samples) and flash frozen and then stored in liquid nitrogen.

Adults for RNA extraction were collected as late-stage nymphs from Wendebachstausee near Göttingen, Germany (3 females, 2 males, N51°28’10.41, E9°56’24.98) in July and August of 2012 and from Erlangen, Germany (3 males, 3 females, N 49°36'35" E 10°59'02") in August 2013. Animals were reared for the final stages in the laboratory to ensure that females were unmated. After reaching maturation, individuals were sacrificed and the gut and one hind leg were removed. Thereafter, the animal was flash frozen and stored in liquid nitrogen. All adults were processed individually.

### RNA extraction and sequencing

We had a total of 17 samples, eleven adults that were processed individually and six ontogenetic stages that were processed by stage. The eleven adult individuals (minus a single leg removed for a DNA sample) and pooled developmental stages were separately homogenized in TriFast (PEQLAB, Erlangen, Germany) using a Minilys homogenizer with the Precellys ceramic kit 1.4/2.8 mm (PEQLAB, Erlangen, Germany). Total RNA was extracted from the 17 samples following the manufacturer’s instructions for TriFast. Total RNA samples were checked for purity and quality using a NanoDrop spectrophotometer (NanoDrop Products, Wilmington, Delaware) and a 2100 BioAnalyzer (Agilent Technologies, Santa Clara, California). If samples had a 260/280 ratio appreciably lower than 2.0, a 230/280 ratio lower than their 260/280 ratio, or severe degradation visible on the Agilent RNA 6000 Pico Assay electropherogram they were not processed further. RNA quantification was done using a NanoDrop spectrophotometer (NanoDrop Products, Wilmington, Delaware) and a Qubit (Thermo Fischer Scientific, Waltham, Massachussetts). To decrease the concentration of ribosomal RNA and to isolate mRNA, a mRNA enrichment was performed using the Dynabeads mRNA Purification Kit (Life Technologies, Carlsbad, California).

Directional, strand specific RNA libraries for Illumina sequencing were prepared with the NEXTflex Directional RNA Seq Kit (dUTP based;Bioo Scientific, Austin, Texas). Libraries were checked for quality using an Agilent High Sensitivity DNA Chip on the 2100 BioAnalyzer. A Qubit 2.0 fluorometer was used to determine the library concentration (Life Technologies, Carlsbad, California). Only libraries with a distinct band at approximately 350bp and a concentration >10nM were deemed high quality and sequenced. After quality check, libraries were sent to the Max-Delbrück-Centrum (Berlin, Germany) for cluster generation and sequencing. Libraries were sequenced on a HiSeq 2000 (Illumina, San Diego, California) to generate 100-bp paired-end reads. All 17 libraries were individually barcoded using NEXTflex RNA-Seq Barcodes from Bioo Scientific (Austin, Texas) and then sequenced at a depth of 4–5 libraries per lane.

### Transcriptome assembly

Reads from all libraries were processed to remove sequencing primers, adaptors, and low quality bases on the 3’ end using Flexbar [38]. Only reads ≥20 bp were retained and after processing we had 74,957,776–93,277,908 reads per sample. Pooled reads from 12 libraries (all 6 ontogenetic stages, 2 males from Göttingen, 3 females from Göttingen, and one male from Erlangen) were assembled together using trinity version r20140717 [39] with the default parameters but specifying the strand specificity of sequencing reads. These 6 adults were chosen for the assembly as they had the highest number of reads. Attempts to include more individuals in the library assembly did not produce significantly better assemblies (data not shown). Trinity produces multiple contigs per isoform cluster (i.e. unigenes). We mapped all of our reads back to the transcriptome using the trinity script align_and_estimate_abundance.pl [40] and then retained only isoforms that accounted for 51% or more of the expression for that contig to reduce redundancy. Additionally, we removed all transcripts less than 600 bp and all transcripts with an FPKM (fragments per kilobase per million fragments mapped) lower than 0.1 as we were uninterested in very low abundance transcripts. This resulting transcriptome was used as a reference for all further analyses. We assessed the completeness of our transcriptome by running BUSCO which looks at completeness of single copy orthologs [41]. For this we used their arthropod single copy ortholog set.

### Transcriptome annotation

The de novo reference transcriptome was compared against the *Drosophila melanogaster* proteome (downloaded from NCBI on November 1^st^ 2013) and Genbank’s non-redundant (nr) database (downloaded from NCBI on July 16^th^ 2014) using the BLASTX algorithm. The transcriptome was first compared with the *D*. *melanogaster* proteome. We filtered out transcripts without hits and these transcripts were then compared with the nr database. Hits with e-values ≤10^−5^ were considered significant. Blast2GO [42] was used to assign Gene Ontology (GO) terms to the transcripts and to identify Interpro domains using InterproScan [43]. GO terms were assigned to transcripts using the *Drosophila melanogaster* blast match if there was one or the nr blast match if there was no *D*. *melanogaster* match. We computed the number of predicted proteins in our transcriptome using the transdecoder package from trinity [44].

### Differential expression analysis

We used the DESeq2 program [45] to determine which transcripts were differentially expressed (DE) during ontogeny. Reads from all 6 developmental stages and 6 adults (3 males from Erlangen and 3 females from Erlangen) were separately aligned to the reference transcriptome using bowtie2 [46] and transcript abundances were calculated for each stage using RSEM [47]. These adults were chosen (rather than the ones used to make the transcriptome) because they were all collected on the same date and from the same location and thus were more standardized than other samples. Alignment and estimation of transcript abundances were done using the Trinity wrapper [40] and we used the gene level output for further analyses. DESeq2 was run using the likelihood ratio test (LRT) with a full model that included stage and sex (as there is strong DE between adult males and females). In this way dispersions were calculated from biologically replicated stages (adults) and these dispersions were used to determine DE genes between stages. We used the LRT test rather than the standard Wald test as our model had multiple terms (sex and stage) and we wanted to test for these simultaneously. All genes that were solely differentially expressed between adult males and adult females (and not between ontogenetic stages) were removed from our list of DE genes. We do not include expression differences between males and females here because another manuscript from our group analyzing sex differences in detail is in preparation. DESEq2 outputs adjusted *p*-values (padj) obtained using the Benjamini-Hochberg procedure [48] and only contigs with a padj <0.001 were classified as differentially expressed. A regularized logarithmic transformation (rlog), which estimates the relationship between mean and variance of the data and transforms the data based on this relationship, was applied to the RSEM expression values. After this, expression values were averaged between all adults (to obtain a single expression value per ontogenetic stage). Differentially expressed genes were clustered using model-based clustering in the R package mclust [49] where 1 to 20 components were simulated and the best number of clusters was estimated based on Bayesian Information Criterion. To place these clusters in a meaningful context we tested for enrichment of functional categories with Gene Ontology (GO) enrichment implemented in Blast2GO using the transcriptome as the reference and a Fisher’s exact test [42]. Afterwards enriched GO terms were reduced to the most specific terms to avoid redundancy.

### Validation of gene expression by semi-quantitative RT-PCR

2 μg of RNA from each sample (6 developmental stages and 6 adults) was used for cDNA synthesis with the “QuantiTect Reverse Transcription Kit” from Qiagen (Venlo, The Netherlands). We amplified nine loci from all 12 cDNA samples used in our differential expression analysis (S3 Table). The amplifications were carried out in a volume of 25 μl of 1X reaction buffer (10X ThermoPol Reaction Buffer/NEB) containing 0.2 μM of each primer, 200 μM of dNTPs (NEB/N0447), and 1 unit Taq Polymerase (NEB/M0267). There were two replicates of each PCR that were performed on two different days using the same protocol (50ng cDNA as template per reaction, 94°C initial denaturation for 4 minutes, a temperature cycle of 94°C for 30 s, 58°C for 40 s and 72°C for 45 s was repeated 30 times, followed by a final extension of 72°C for 10 minutes). PCR products were run on a 3% agarose gel with a 100 bp ladder (Roth/ T834.1) and gel images were analyzed with Syngene´s GeneTools Analysis Software using the ladder concentrations as a standard.

## Results

### Transcriptome assembly

We sequenced RNA from six developmental stages and eleven adults. Our 17 libraries had between 38,883,144 and 95,132,876 reads per library (Average 69,137,156) after quality trimming and filtering.

Using Trinity we generated a reference transcriptome from our reads. The initial transcriptome output by Trinity contained 1,564,070 contigs with an N50 of 424 bp (Table 1). As we were not interested in lowly expressed genes, we removed any contig that had an FPKM of less than 0.1. To lower redundancy, we removed all isoforms that accounted for less than 51% of the reads per trinity ‘gene’ thus ensuring a maximum of one isoform per trinity ‘gene’. After filtering, we retained 444,844 contigs with an N50 of 515 bp. Finally, after filtering out any contigs less than 600 bp we were left with 76,522 contigs with an N50 of 1,262 bp. Transdecoder indicated that we had 23,562 transcripts in our transcriptome with at least partial open reading frames. Of these, 3,614 did not have blast matches indicating that they are novel proteins. Transcripts without detectable open reading frames may be protein coding or non-coding RNAs.

**Table 1:** Assembly statistics for the full and reduced transcriptome.

	Full	Reduced (Isopct ≥ 51, FPKM ≥ 0.1, length ≥ 600 bp)
Number of transcripts	1,564,070	76,522
Total length of transcripts (bp)	212,567,026	92,204,341
Mean transcript length	478	1,205
Median transcript length	332	885
N50	424	1,262
GC%	41.78%	41.44%

Isopct—percentage of transcripts abundance over its parent gene’s abundance, FPKM—Fragments per kilobase of transcript per million mapped reads.

We ran BUSCO to check for the presence of 2,675 arthropoda single copy orthologs. Of these, we found 1,750 complete, 184 fragmented, and 741 missing. Of the 1,750 complete orthologs, 1,513 were single copy and 237 were duplicated.

### Transcriptome annotation

We used BLASTX and Blast2GO to annotate our reference transcriptome. We first used a BLASTX search against the *Drosophila* proteome finding 15,715 transcripts with matches with an evalue of 10^−5^ or lower. Unmatched contigs were blasted against the non-redundant protein database from NCBI finding an additional 12,891 transcripts with matches. Unmatched transcripts are likely shorter (shorter transcripts are less likely to match genes in the database) or encode novel proteins. Transcripts without BLASTX matches were almost half the length (980 bp) of transcripts with matches (1582 bp). Given the dearth of genomic resources for hemimetabolous insects, transcripts without matches are also likely to encode novel proteins. Using the interproscan function in Blast2GO, we were able to assign protein signatures to 34,414 contigs. Finally, using the results from both interproscan and BLASTX, 19,289 contigs were annotated with at least one GO term.

### Differential expression

**Fig 2:**
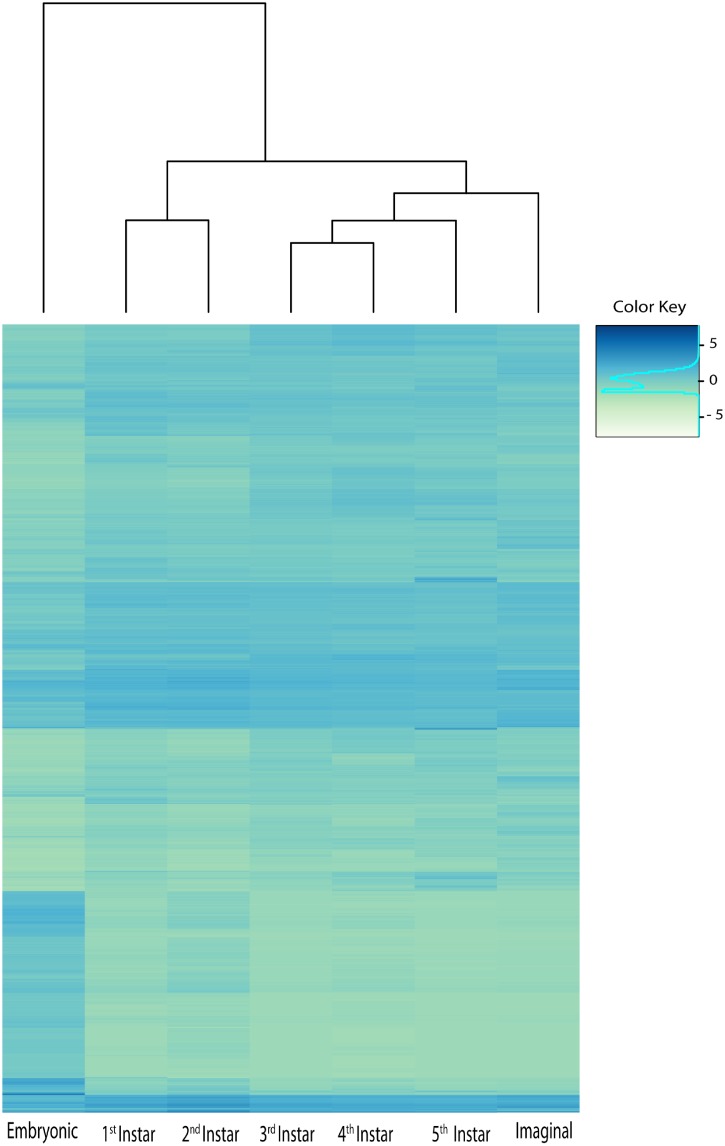
Heatmap of the 5,150 differentially expressed transcripts. FPKM values have been log transformed and scaled. Color intensity indicates expression level. Similarity between stages with hierarchical clustering is shown above the heatmap.

We were interested in examining changes in expression across development. Using an FDR cut-off of P< 0.001, 5,150 transcripts were found to be differentially expressed between at least 2 of the stages (S1 Table). Clustering of different developmental stages across these transcripts revealed that the embryonic stage was strongly differentiated from all instar stages in terms of their gene expression patterns (Fig 2). The instar stages were split by age with the first and the second instars grouping together and the last three instars grouping together with the imaginal stage (Fig 2).

**Fig 3:**
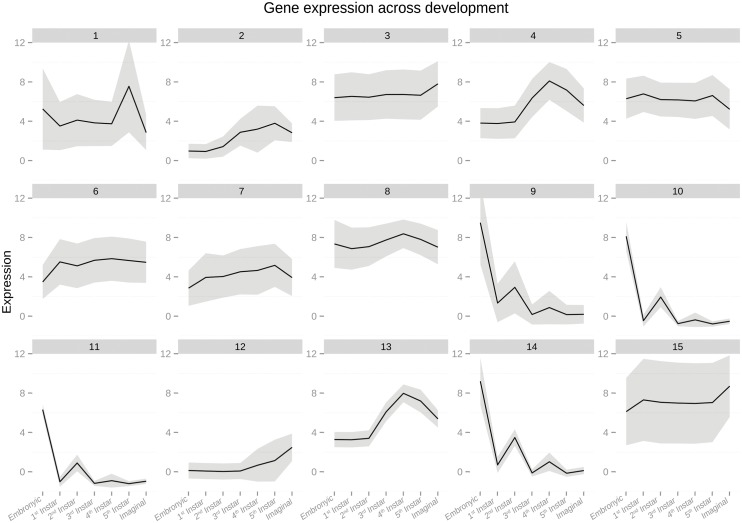
Plots of the 15 gene expression clusters. Plots of the 15 gene expression profiles with the mean expression highlighted. The black line indicates the average expression values while the grey shaded areas indicate maximum and minimum expression values of genes in the cluster.

Model-based clustering indicated that the best-fit model had 15 clusters (plotted in Fig 3). Some clusters showed similar expression patterns but with different magnitudes (i.e. clusters 9, 10, 11, and 14). We identified 9 different gene expression profiles. Cluster 1, containing 166 transcripts, showed strongly increased expression in the 5^th^ instar and slightly increased expression in the embryonic stage. Clusters 2 and 12, containing 358 and 202 transcripts respectively, both had the general pattern of decreased expression in early development and increased expression in later stages but the patterns were not identical. Cluster 3, containing 517 transcripts, showed increased expression in the imaginal stage. Clusters 4 and 13, containing 288 and 189 transcripts, peaked in the 4^th^ instar. Clusters 5 and 7, with 486 and 368 transcripts respectively, were weakly downregulated in the embryonic and imaginal stages. Cluster 6, containing 505 transcripts, showed decreased expression in the embryonic stage and a slight decrease in the second instar. Cluster 8, with 410 transcripts showed peak expression in the 4^th^ instar with elevated expression in the embryonic stages as well. Clusters 9, 10, 11, and 14, containing 75, 412, 617, and 315 transcripts, showed strongly increased expression in the embryonic stage with an additional peak in the 2^nd^ instar stage. Finally, cluster 15, with 242 transcripts showed decreased expression in the embryonic stage but increased expression in the imaginal stage.

#### GO enrichment

Using Fisher’s Exact Test with a FDR correction, enriched GO terms for each cluster were determined. Between 1 (cluster 12) and 151 (cluster 5) enriched GO terms were found for each cluster after reducing the terms to their most specific (i.e. highest GO level, S2 Table).

Cluster 1, with increased expression in the last instar, was enriched for multiple processes relating to the cuticle. Cluster 2, which had generally decreased expression in early development, was not enriched for any processes. Cluster 12, which showed a superficially similar pattern to cluster 2, was enriched for serine-type endopeptidase activity. Cluster 3, which had increased expression in the imaginal stage, was enriched for many terms involved in proteolysis and metabolism. Cluster 4, which showed higher expression in the 4^th^ instar, was enriched for multiple terms relating to mitosis. Cluster 13, which showed the same pattern, was enriched for multiple terms relating to the synthesis of neurotransmitters. Clusters 5 and 7 showed decreased expression in the embryonic and adult stages. Cluster 5 was enriched for the largest number of terms of any cluster, and contained multiple terms for various developmental processes (ex: limb development, organ formation) as well as cellular differentiation and sex differentiation. Cluster 7 was not enriched for any terms. Cluster 6 showed decreased expression in the embryonic stage and was enriched for terms that relate to dealing with foreign compounds and oxidative stress. Cluster 8 showed upregulation in the 4^th^ instar with a slight upregulation in the embryonic stage as well. Cluster 8 was enriched for multiple developmental signaling pathways (JAK-STAT and Wnt), several terms relating to gamete development, meiosis, and nervous system development. Clusters 9, 10, 11, and 14 showed strongly increased transcription in the embryonic stage and weakly increased transcription in the second instar. Cluster 9 was enriched for terms relating to ribosomes and cluster 10 was enriched metabolic activity and other housekeeping processes. Cluster 11 was enriched with terms relating to transcription. Cluster 14 was enriched for several terms relating to ribosomes, transcription, mitosis, and chromatin remodeling. Cluster 15, which showed decreased expression in the embryonic stage and increased expression in the imaginal stage was enriched for multiple terms relating to muscle activity and vision.

### Validation of differential expression results

We validated the results from our differential expression analysis using semi-quantitative PCR on 9 genes (S4 Table). This analysis was in agreement with the patterns observed in our differential expression analysis (S4 Table, S1 and S2 Figs).

## Discussion

In this study, we assembled a de novo transcriptome spanning 7 ontogenetic stages from the embryonic to the imaginal of the grasshopper, *Chorthippus biguttulus*. We then performed a differential expression analysis to observe how expression patterns change through ontogeny and to identify major processes happening at each stage. We found 9 different patterns of gene expression (across 15 clusters) that provide information about the different types of processes involved in the different stages of development. The expression patterns reveal potentially key pathways and processes that underlie the developmental pathway that progresses from embryo to imago in Acridid grasshoppers and thus hemimetabolous insects. Additionally, the transcriptome we have developed represents a new resource that can be used in future studies. Below we discuss a subset of our clusters that we believe best illuminate the developmental process.

### Transcriptome

Our transcriptome contains 76,522 contigs. Approximately 1/3 of these have open reading frames (23,562) including 3,614 potentially novel proteins. BUSCO analysis using the arthropod single copy orthologs indicated that our transcriptome is 65% complete (1,750 complete orthologs out of 2,675). This is similar to results from other arthropods such as the freshwater crayfish [64% complete, 50], the western tarnished plant bug [74% complete, 51], the grasshopper *Shirakiacris shirakii* [63% complete, 52], and the pygmy grasshopper [72% complete, 53], although other studies have reported higher values.

### Embryonic specific processes

In our data, four clusters showed strong up-regulation of transcription in the embryonic stage (Clusters 9, 10, 11, and 14) and two clusters showed weak up-regulation (Clusters 1 and 8). The strongly upregulated clusters were enriched in GO terms related to ribosomes. This is concordant with the large amount of gene expression that goes on during embryonic development. Regulating ribosomal numbers helps to control cellular growth and proliferation during development in *D*. *melanogaster* [54] and a similar mechanism may be occurring in *C*. *biguttulus*. The strongly upregulated clusters were also enriched in GO terms relating to transcription and translation. To determine if any of these genes relating to transcription were specifically involved in embryonic development, we examined the transcript annotations for the 5 enriched categories linked to gene expression in cluster 11 (holo TFIIH complex, carbox-terminal domain protein kinase complex, gene expression, RNA polymerase II carboxy-terminal domain kinase activity, and promoter clearance from RNA polymerase II promoter). We found a large number of genes involved in developmental processes and discuss a subset here. The ubiquitin-conjugating enzyme effete (*eff*) is required for germline stem cell maintenance [55] as well as neural development [56] in *Drosophila melanogaster*. Nuclear transcription factor YB is one of three subunits that comprise nuclear transcription factor Y, which functions in the JNK pathway during thoracic development in *D*. *melanogaster* [57] and is necessary for cell death and differentiation during eye development in *D*. *melanogaster* [58]. The transcription factor cubitus interruptus (*Ci*) interacts with the Hedgehog pathway as both an activator and repressor of different Hedgehog target genes and is necessary for limb development in *D*. *melanogaster* [59]. The casein kinase Discs overgrown (*Dco*) is a component of the Hippo pathway, which is a highly conserved pathway that regulates organ size [60]. Although we do not get enrichment of any of these known developmental pathways in the strongly upregulated clusters, some of the genes involved in these pathways are clearly upregulated during embryonic development in *C*. *biguttulus*.

Although some of the genes involved in these pathways were strongly upregulated during embryonic development others were weakly upregulated. Cluster 8 was enriched for both the JAK-STAT cascade and regulation of the Wnt receptor signaling pathway. The JAK-STAT cascade controls the formation of many different tissues and can regulate multiple processes from cell shape to tissue folding [61]. Wnt is involved in posterior development and segmentation [62]. Additionally, cluster 8 was enriched in numerous GO terms relating to development such as neuron differentiation, reproductive structure development, eye morphogenesis, and epithelial cell differentiation among others (S1 Table). Cluster 1, which was also weakly upregulated in the embryonic stage, was enriched in GO terms relating to sclerotization (the process by which the cuticle becomes rigid) including structural constituent of chitin-based cuticle and catechol oxidase activity [catechols are oxidized to form highly reactive tanning agents in the sclerotization process, 63].

Taken together, these results suggest that known developmental pathways are indeed active in *C*. *biguttulus* and that genes involved in these pathways may show either strong upregulation in the embryonic stage or only moderate upregulation. This probably depends on whether the gene is expressed earlier or later in the particular pathway and how specific its expression pattern is (i.e. expressed in the entire embryo versus only in certain developing tissues).

### The 4^th^ instar

Several clusters showed strong up-regulation of gene expression in the 4^th^ instar indicating that there are stage specific processes occurring here. Clusters 4 and 13 both showed patterns of strong increases in gene expression in the second half of development peaking in the 4^th^ instar. Cluster 4 was enriched in GO terms involving mitosis and metabolism whereas cluster 13 was enriched in terms relating to the synthesis of various biogenic amines (serotonin, dopamine, and histamine). These substances can act as neurotransmitters, modulaters of adenylate cyclase, or regulators of reproductive function [64, 65]. A study in *Drosophila melanogaster* suggests that biogenic amines may regulate the metabolism of juvenile hormone, which plays a role in molting during development and reproduction in adults [65]. Juvenile hormone titers change drastically during the last instars in *Locusta migratoria*, a closely related Acridid [64]. Up-regulation of biogenic amines in this developmental stage may be correlated with changing levels of juvenile hormone or it may serve another purpose. Further work in Orthoptera is necessary to make the distinction.

### The 5^th^ instar (sub-adult)

The 5^th^ instar is the last stage before reaching the imago stage and is also the last stage before the wings are fully developed. We found genes involved in wing development as well as development of the adult chitin exoskeleton upregulated in this stage. Cluster 1 was strongly upregulated in the 5^th^ instar stage but only weakly upregulated in the embryonic stage. It was enriched in several GO terms associated with chitin such as: structural constituent of chitin-based cuticle, catechol oxidase activity, and chitin binding. We examined the annotation of the transcripts in these categories since adult chitin is initiated during this stage. *CHT5* and *CHT8* are chitinases that are expressed in all stages, but most highly in the 5^th^ instar. Data from *Locusta migratoria* suggests that CHT5 is involved in chitin metabolism during cuticle turnover [66]. Also present is *dusky-like*, which is important for bristle development and wing hair morphogenesis in *D*. *melanogaster* [67, 68], this is consistent with the final wing morphogenesis that occurs during *C*. *biguttulus* 5^th^ instars. Finally, *TwdlE*, which is part of a family of cuticle proteins (The Tweedle Family) and is important for larval and pupal body shape in *D*. *melananogaster* [69], was upregulated in the 5^th^ instar. These results indicate that many of the genes activated during the 5^th^ instar might be involved in development of the adult cuticle. These results suggest that cuticle development and wing formation in *C*. *biguttulus* and *D*. *melanogaster* utilize similar mechanisms.

### Imago specific processes

We found genetic evidence for adult specific processes, like reproduction, mating, and aging, but also additional processes such as immune system regulation. Cluster 3 was enriched in transcripts relating to both aging and determination of adult lifespan. We examined the annotation of genes making up both of these enrichment groups. One of these genes, *Cct1*, is required for ovary development and oogenesis in *D*. *melanogaster* [70]. We also found takeout (*To*), which is involved in circadian rhythms, male courtship behavior, and feeding behavior in adult *D*. *melanogaster* [71, 72]. Takeout has also been implicated in the attraction/repulsion responses of gregarious/solitary forms of the closely related Acridid *Locusta migratoria* [73]. Since takeout is primarily expressed in the antennae it has been suggested that it may function in olfactory perception. This is concordant with the strong CHC communication system recently discovered in adult *C*. *biguttulus* [74] making takeout a potential candidate gene for olfactory communication in *Chorthippus*. An aldehyde oxidase (*AOX1*), also upregulated in adults, is also expressed only in antennae and is thought to degrade aldehyde odorant compounds for signal termination [75]. Cluster 3 was also enriched for genes involved in peptidase inhibitor activity. Most of the genes were annotated as serpins, which are serine/cysteine peptidase inhibitors. Serpins are necessary for innate immune system regulation [76]. It is possible that adult *C*. *biguttulus* are exposed to more pathogens and xenobiotic compounds than developing embryos or nymphs as they interact more with the external environment. Finally, we also found genes responsible for reducing oxidative stress: catalase (*cat*) and superoxide dismutase (*SOD*), both of which reduce oxidative stress and thus affect lifespan [77, 78].

We have generated a new resource for Acridid grasshoppers and used this to examine ontogenetic changes in gene expression across the entire transcriptome. Most developmental studies concentrate solely on embryonic development, but we found dynamic changes in gene expression throughout the instar stages as well. In particular, we found that some instar stages undergo greater differentiation than others (instars 4 and 5); we suggest that this new finding be considered in future studies of post-embryonic development. Our results indicate that post-embryonic development is an uneven process (i.e. not continuous) and highlight the importance of looking at the whole of ontogeny. Although our work gives insight into processes that may be occurring in later stages, targeted studies will be necessary to confirm our findings.

## Supporting information

S1 Fig Normalized expression from RNAseq and semi-quantitative RT-PCR. In each graph the blue line indicates the results from the semi-quantitative RT-PCR and the red line indicates results from our RNAseq data. All data sets have been normalized for comparison using the equation Z = (X-μ) / σ where a μ of 10 and the un-normalized variance of the data set were used. A. c1130743_g1_i1 B. c1102461_g1_i2 C. c1042330_g1_i1 D. c1463247_g1_i1 E. c1112983_g3_i1 F. c1028189_g1_i1 G. c1080794_g3_i1 H. c1071364_g1_i1 I. c1089044_g3_i3.
(PDF)

S2 Fig Correlation of gene expression results between RNAseq and RT-PCR. Correlation of gene expression results obtained from semi-quantitative RT-PCR and RNAseq for 9 genes and 12 samples.
(PDF)

S1 Table Differentially expressed transcripts. Listed are the annotation, clustering results, and GO terms for each differentially expressed transcript.
(XLSX)

S2 Table Gene Ontology results. Results of GO enrichment analyses for all clusters.
(XLSX)

S3 Table Contigs used for semi-quantitative RT-PCR validation. (XLSX)

S4 Table Results of semi-quantitative RT-PCR validation. The top table is the raw read counts from the RNAseq data. The bottom table is the estimated ng of product using the GeneTools software. All values in the bottom table are averaged over two replicate PCR reactions for each primer-sample combination.
(XLSX)
