# Supplementary figures and images for: Transcriptome profiling of ontogeny in the acridid grasshopper Chorthippus biguttulus
Source: PLoS One. 2017 May 17;12(5):e0177367. doi: 10.1371/journal.pone.0177367 (PMC5435247; doi:10.1371/journal.pone.0177367)

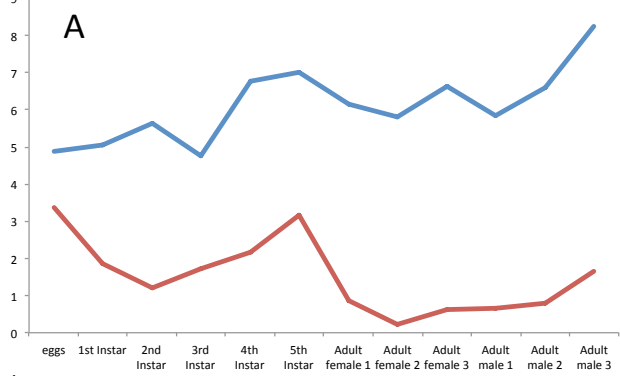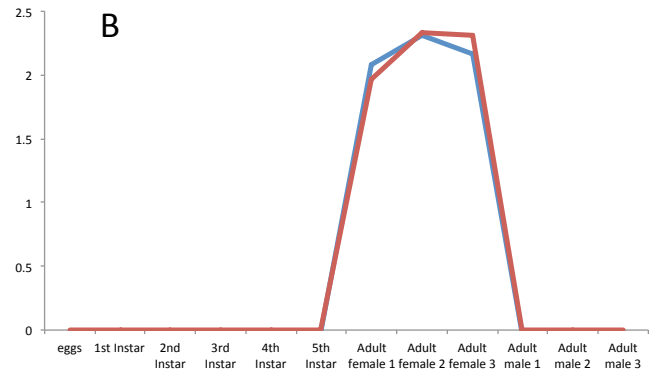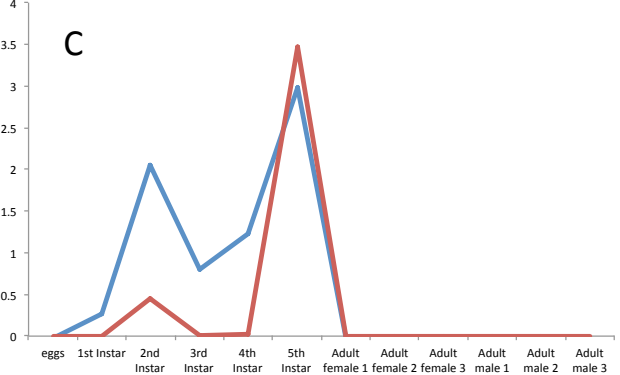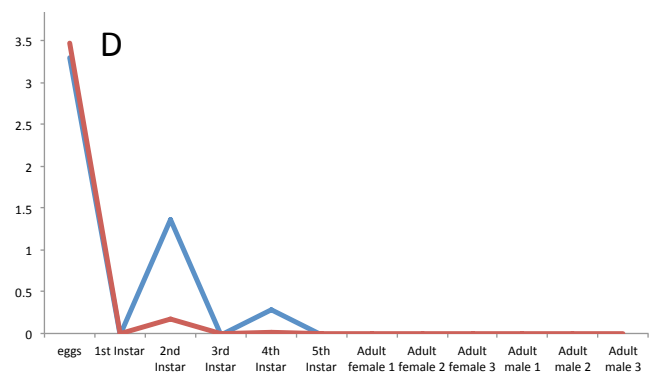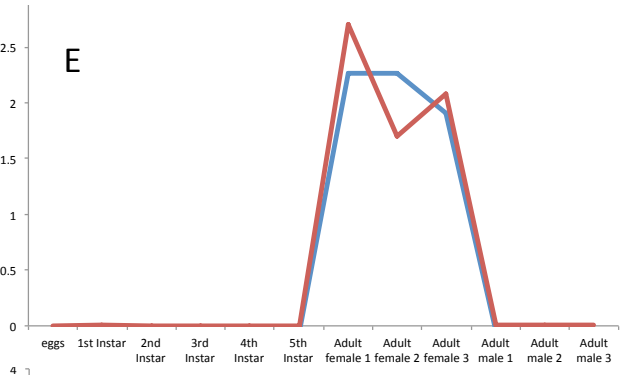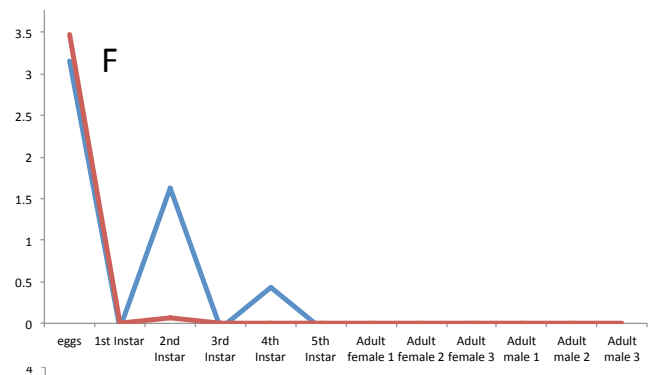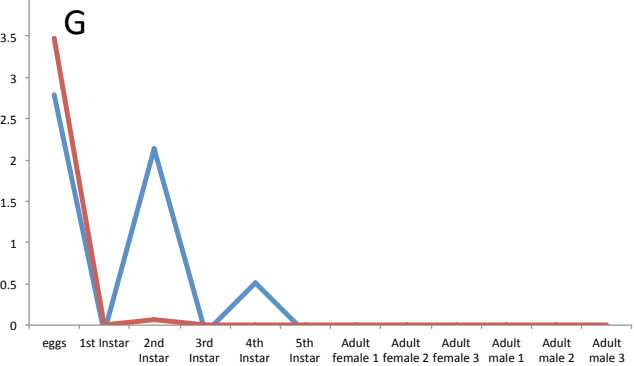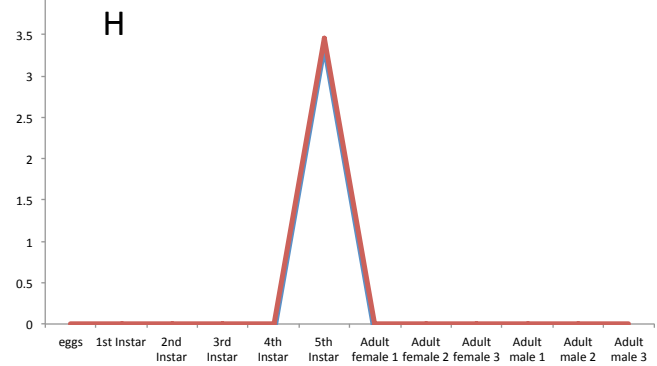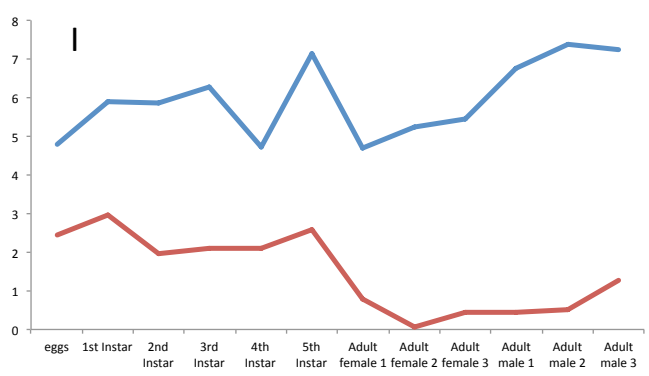

Supplement: S1 Fig — In each graph the blue line indicates the results from the semi-quantitative RT-PCR and the red line indicates results from our RNAseq data. All data sets have been normalized for comparison using the equation Z = (X-μ) / σ where a μ of 10 and the un-normalized variance of the data set were used. A. c1130743_g1_i1 B. c1102461_g1_i2 C. c1042330_g1_i1 D. c1463247_g1_i1 E. c1112983_g3_i1 F. c1028189_g1_i1 G. c1080794_g3_i1 H. c1071364_g1_i1 I. c1089044_g3_i3. (PDF) [file pone.0177367.s001.pdf]

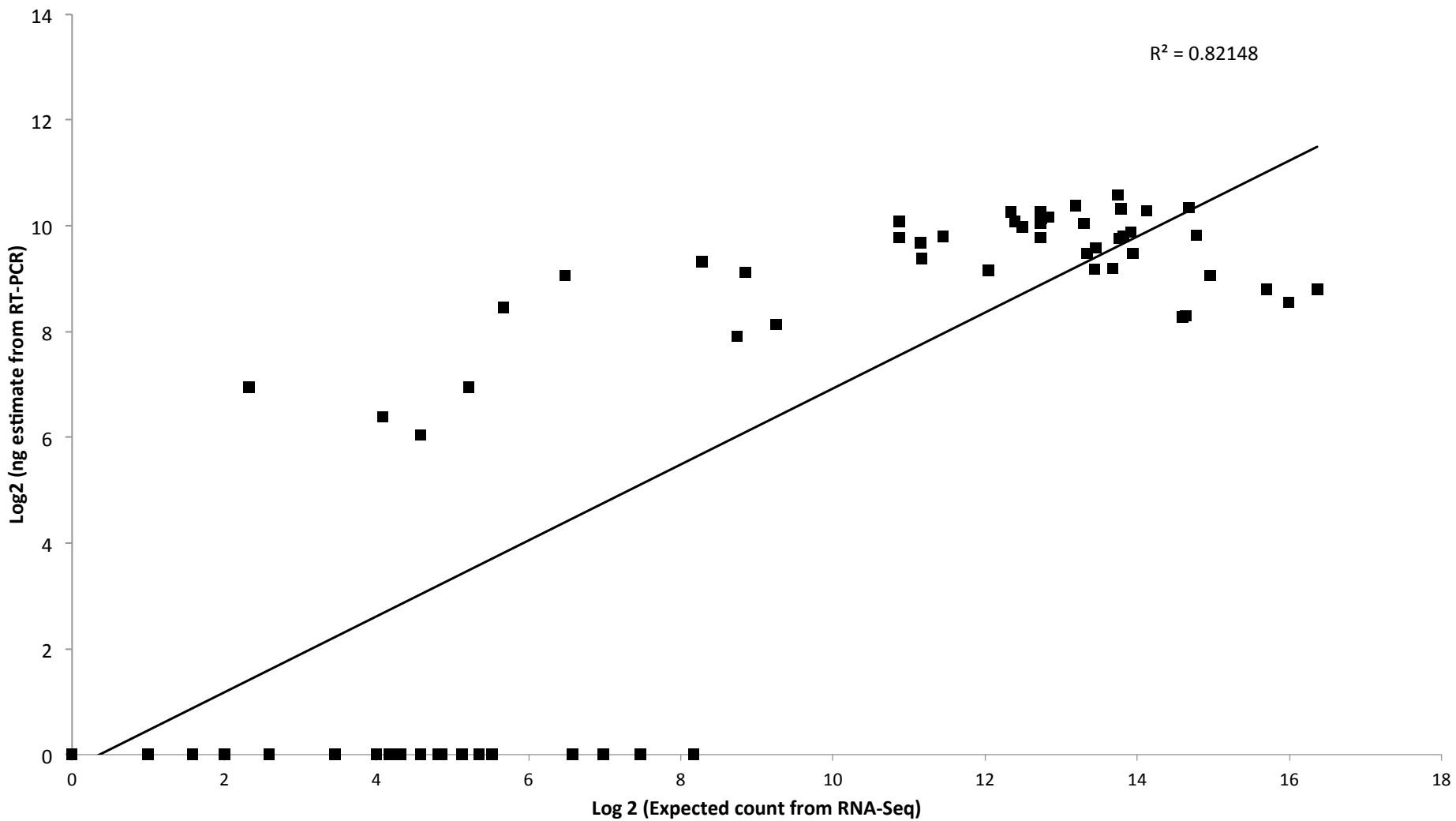

Supplement: S2 Fig — Correlation of gene expression results obtained from semi-quantitative RT-PCR and RNAseq for 9 genes and 12 samples. (PDF) [file pone.0177367.s002.pdf]
